# Generalized tuberculosis in HIV-infected patients with AIDS

**DOI:** 10.1186/1758-2652-13-S4-P195

**Published:** 2010-11-08

**Authors:** VN Zimina, IA Vasilieva, AV Kravchenko, FA Batyrov

**Affiliations:** 1 Central TB Research Institute under Russian Academy of Medical Sciences, Moscow, Russian Federation; 2 Central TB Research Institute, Russian Academy of Medical Sciences, Moscow, Russian Federation; 3 Central Research Institute of Epidemiology under Rospotrebnadzor, Moscow, Russian Federation; 4 TB Clinical Hospital #7, Moscow, Moscow, Russian Federation

## Purpose of the study

To estimate peculiarities of diagnosis and clinical course of generalized TB in HIV patients with AIDS who received treatment in TB Hospital #7, Moscow in 2006-2009.

## Methods

94 cases (47,9%) of generalized TB have been analyzed. Most of them are men (82,9%), the mean age of the patients being 31,2±7,02. The average period from HIV detection to TB diagnosis is 5,7±2,96 years.

## Results

In 62 cases (66%) the clinical TB symptomatology developed quite fast, in 2,2±1,9 months on average. An acute beginning was seen in 32 cases (34%). 44 patients (46,8%) had both TB and other secondary diseases. The CD4 lymphocyte mean level was 91 cells/mm^3^. M. tuberculosis was found in 52 cases (55,3%): in the sputum analyses of 26 patients (27,7%) and in 26 more analyses of other biological materials (such as exudates, urine, feces, liquor, bioptic and surgical material). Among 29 patients tested for TB drug resistance 13 (44,8%) proved to be multidrug-resistant TB cases. The chest X-ray examination showed intrathoracic lymphoadenopathy in 68,1% cases, interstitial dissemination in 29,7% cases and only 7,5% cases revealed disintegration of tissue. 77 patients (81,9%) underwent different surgical interventions for the purpose of diagnosis or treatment, namely: diagnostic laparoscopy (16 cases), curative laparotomy (22), mediastinoscopy with intrathoracic lymph node biopsy (4), pleura biopsy (9), debridement of a peripheral lymph node (17), pericardial microdrainage (6), pleural cavity drainage (2), orchectomy (1). While examining the received diagnostic material morphological markers of TB inflammation were found in 56 cases (59,6%). Among most often discovered extrapulmonary localizations are abdominal involvement (53,3%), nodal involvement (27,7%), meningoencephalitis (15,9%), pericarditis (15,9%). Involvement of more than 3 systems was diagnosed in 25 patients (26,6%). Specific TB treatment included 4 to 6 drugs, the follow-up period for complete treatment was 6 months. Treatment results: cured - 36 cases (38,3%), defaulters - 34 cases (36,1%), died - 24 cases (25,5%).

## Conclusions

Thus, providing treatment and diagnosis to patients with multiple-localization TB is complicated and requires an interdisciplinary approach including different surgical methods of diagnosis and treatment. The CD4 lymphocyte level lower than 100 cells/mm^3^ before treatment increases significantly the probability of an unfavorable outcome of generalized TB.

